# Dexamethasone Acetate‐Loaded PLGA Nanospheres Targeting Liver Macrophages

**DOI:** 10.1002/mabi.202400411

**Published:** 2024-11-29

**Authors:** Barbora Boltnarova, Anna Durinova, Lenka Jandova, Stanislav Micuda, Otto Kucera, Ivona Pavkova, Miloslav Machacek, Ivana Nemeckova, Marek Vojta, Jan Dusek, Maria Krutakova, Petr Nachtigal, Petr Pavek, Ondrej Holas

**Affiliations:** ^1^ Department of Pharmaceutical Technology Faculty of Pharmacy in Hradec Kralove Charles University Akademika Heyrovskeho 1203 Hradec Kralove 50005 Czech Republic; ^2^ Department of Pharmacology and Toxicology Faculty of Pharmacy in Hradec Kralove Charles University Akademika Heyrovskeho 1203 Hradec Kralove 50005 Czech Republic; ^3^ Department of Pharmacology Faculty of Medicine in Hradec Kralove Charles University Simkova 870 Hradec Kralove 50003 Czech Republic; ^4^ Department of Physiology Faculty of Medicine in Hradec Kralove Charles University Simkova 870 Hradec Kralove 50003 Czech Republic; ^5^ Department of Molecular Pathology and Biology Military Faculty of Medicine University of Defence Trebesska 1575 Hradec Kralove 50001 Czech Republic; ^6^ Department of Biochemical Sciences Faculty of Pharmacy in Hradec Kralove Charles University Akademika Heyrovskeho 1203 Hradec Kralove 50005 Czech Republic; ^7^ Department of Biological and Medical Sciences Faculty of Pharmacy in Hradec Kralove Charles University Akademika Heyrovskeho 1203 Hradec Kralove 50005 Czech Republic; ^8^ Department of Physics Faculty of Science University of Hradec Kralove Rokitanskeho 62 Hradec Kralove 50003 Czech Republic

**Keywords:** biodegradable nanoparticles, glucocorticoids, liver inflammation, macrophages, PLGA nanospheres

## Abstract

Glucocorticoids are potent anti‐inflammatory drugs, although their use is associated with severe side effects. Loading glucocorticoids into suitable nanocarriers can significantly reduce these undesirable effects. Macrophages play a crucial role in inflammation, making them strategic targets for glucocorticoid‐loaded nanocarriers. The main objective of this study is to develop a glucocorticoid‐loaded PLGA nanocarrier specifically targeting liver macrophages, thereby enabling the localized release of glucocorticoids at the site of inflammation. Dexamethasone acetate (DA)‐loaded PLGA nanospheres designed for passive macrophage targeting are synthesized using the nanoprecipitation method. Two types of PLGA NSs in the size range of 100–300 nm are prepared, achieving a DA‐loading efficiency of 19 %. Sustained DA release from nanospheres over 3 days is demonstrated. Flow cytometry analysis using murine bone marrow‐derived macrophages demonstrates the efficient internalization of fluorescent dye‐labeled PLGA nanospheres, particularly into pro‐inflammatory macrophages. Significant down‐regulation in pro‐inflammatory cytokine genes mRNA is observed without apparent cytotoxicity after treatment with DA‐loaded PLGA nanospheres. Subsequent experiments in mice confirm liver macrophage‐specific nanospheres accumulation following intravenous administration using in vivo imaging, flow cytometry, and fluorescence microscopy. Taken together, the data show that the DA‐loaded PLGA nanospheres are a promising drug‐delivery system for the treatment of inflammatory liver diseases.

## Graphical abstract

This study presents PLGA nanospheres loaded with dexamethasone acetate (DA), achieving a sustained release of DA over 3 days. Nanospheres loaded with DA demonstrate an anti‐inflammatory effect in vitro and an effective targeting in liver macrophages after intravenous administration in vivo. These findings suggest a promising approach for liver‐targeted anti‐inflammatory treatment.

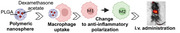

## 1 Introduction

Hepatic inflammation is a serious health condition that is caused by multiple factors, including viral infections, alcohol consumption, autoimmune disorders, or drug‐induced liver injury. If the hepatic inflammation progresses into the chronic stage, it often leads to fibrosis, cirrhosis, and even liver failure.^[^ ^1^, ^2^ ^]^

Macrophages (among the multiple cell populations in the liver) play an essential role in developing hepatic inflammation.^[^ ^3^ ^]^ Kupffer cells, resident macrophages located within the liver sinusoid, are responsible for maintaining hepatic homeostasis. Their main task is to remove blood debris, pathogens, and other foreign particles from the bloodstream. In the case of hepatic injury or inflammation, the liver macrophage population is increased by infiltrated monocyte‐derived macrophages.^[^ ^4^ ^]^ Macrophages can polarize into several subtypes, including pro‐inflammatory (M1) macrophages and anti‐inflammatory (M2) macrophages.^[^ ^5^ ^]^ M1 macrophage polarization is activated by interferon‐gamma or lipopolysaccharide (LPS) with subsequent release of various inflammatory cytokines such as tumor necrosis factor‐alpha (TNF‐α), interleukin (IL) 1 or IL‐6. M2 macrophages encompass several subphenotypes M2a‐d characterized by the production of anti‐inflammatory cytokines such as IL‐10 and IL‐4.^[^ ^5^, ^6^, ^7^ ^]^

Glucocorticoids (GCs) are one of the pivotal pharmacological interventions with a potent anti‐inflammatory activity that can induce a polarization switch toward anti‐inflammatory phenotypes. Such pharmacologically induced change in macrophage polarization could be beneficial in the treatment of liver inflammation.^[^ ^6^ ^]^ Despite the unquestionable therapeutic benefits of GCs, their use is often associated with a considerable number of various side effects. Glucocorticoid receptor (GR) stimulation in the liver significantly impacts macrophages and hepatocytes.^[^ ^8^ ^]^ Upon GC binding to GR within macrophages, nuclear factor‐κB activity is repressed, leading to decreased production of inflammatory cytokines IL‐6, IL‐1β, and TNF‐α.^[^ ^8^, ^9^, ^10^ ^]^ However, GR activation in hepatocytes stimulates gluconeogenesis, fatty acid synthesis, and cholesterol transport in the liver.^[^ ^8^ ^]^ Therefore, conventional GCs dosing with nonspecific distribution into all liver cell types is burdened by undesirable side effects caused predominantly by triggering metabolic pathways within hepatocytes.^[^ ^11^, ^12^ ^]^

Utilizing a suitable nanoparticle‐based delivery system for targeting GCs specifically into macrophages can enhance the safety and efficacy of GC therapy.^[^ ^13^ ^]^ Hepatic macrophages can be effectively targeted via passive targeting by fine‐tuning the formulation's physicochemical properties. Hydrophobic and negatively charged nanoparticles within the 100–300 nm size range are preferably taken up by hepatic and spleen macrophages after intravenous (i.v.) administration.^[^ ^14^, ^15^ ^]^ In our previous report, we developed polylactic‐co‐glycolic acid (PLGA) nanospheres (NSs) with optimized physicochemical properties targeting specifically macrophages.^[^ ^15^ ^]^ The PLGA NSs are biodegradable polymeric drug delivery systems suitable for various active substances, including small lipophilic molecules such as GCs. PLGA is a proven biocompatible and fully biodegradable material that is approved for human use. The advantages of drug loading into PLGA nanoparticles include prolonged drug release, protection of active substances, and the possibility of site‐specific distribution.^[^ ^13^, ^16^ ^]^

The well‐known synthetic GC dexamethasone is a suitable candidate for treating hepatic inflammation. Its anti‐inflammatory activity is 25 times greater than hydrocortisone.^[^ ^17^ ^]^ However, loading dexamethasone into polymeric nanoparticles is challenging and inefficient because of its physicochemical properties (such as logP = 1.8), which results in low loading efficiency.^[^ ^18^, ^19^, ^20^ ^]^ Consequently, its derivative with increased lipophilicity, dexamethasone acetate (DA), was selected as a suitable candidate for loading into PLGA NSs.^[^ ^21^ ^]^

To achieve the desired anti‐inflammatory effect, we aim to optimize the nanoformulation by adhering to specific criteria. These criteria include hydrophobicity, a negative surface charge, stability of the prepared NSs, and a particle size between 100 and 200 nm. Together, these properties facilitate macrophage uptake and allow for sterile filtration. Additionally, the NSs should exhibit adequate GC loading efficiency and capacity to ensure sufficient delivery to macrophages for initiating the desired anti‐inflammatory effect. The formulation should also demonstrate specificity for macrophages while minimizing accumulation in hepatocytes. Finally, the nanocarriers should be suitable for i.v. administration.

In this study, we prepared DA‐loaded PLGA NSs suitable for i.v. administration, which effectively suppresses inflammation in macrophages in vitro. After the i.v. administration, effective and specific accumulation in the liver macrophages was shown. This research demonstrates the promising potential of PLGA formulations for advanced therapy of liver inflammation.

## 2 Result

### 2.1 Characterization of DA‐loaded NSs

**Figure 1:**
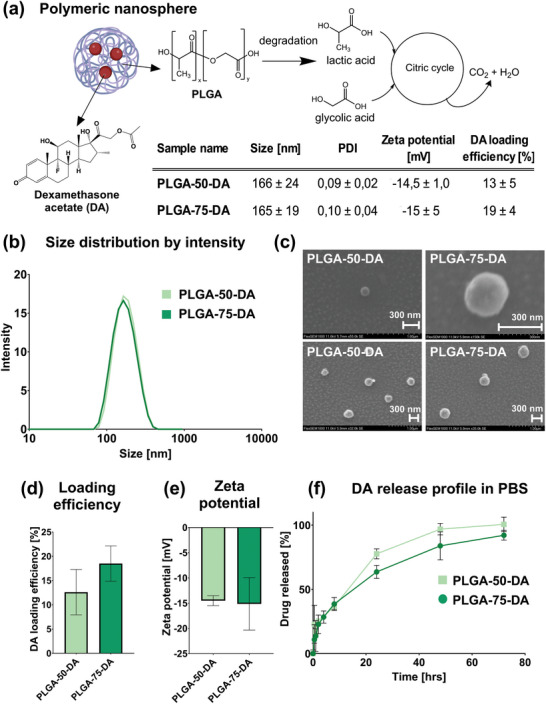
Characterization of dexamethasone acetate (DA)‐loaded PLGA nanospheres (NSs) a) Schematic diagram of prepared biodegradable PLGA NSs and overview of NSs characterization; b) Size distribution by the intensity of DA‐loaded NSs as determined using Zeta Sizer; c) SEM pictures of DA‐loaded NSs, scale bar 300 nm d) Drug loading efficiency of NSs measured by HPLC; e) Zeta potential measured by dynamic light scattering method; f) DA release profile of DA‐loaded NSs in phosphate buffered saline (PBS) pH 7.4 over 72 h. *n* = 3.

Biodegradable NSs loaded with DA were prepared using the nanoprecipitation method. Two different PLGA copolymers were used to prepare NSs with varying properties (**Figure** **1**a). PLGA 75 with acid termination, a ratio between lactic acid and glycolic acid of 3:1, is a more hydrophobic material compared to PLGA 50 with ester termination and an equimolar ratio between lactic and glycolic acid. Both polymers have a molecular weight of 17 000 g mol^−1^. The size of both formulations reached 150 nm (Figure 1b). The size reported in this study is hydrodynamic diameter as dynamic laser scattering is a granulometric method of choice. All tested formulations were monodispersed with a polydispersity index under 0.2. The size and spherical shape were confirmed by scanning electron microscopy (SEM) (Figure 1c). The highest drug loading efficiency (DLE%) of DA (19 ± 4%) was achieved by using PLGA 75 (Figure 1d). Zeta potential of formulations based on PLGA 50 and PLGA 75 was −14.5 ± 1.0 and −15 ± 5 mV, respectively (Figure 1e). The DA release profile from PLGA NSs in pH 7.4 is distinctly biphasic, characterized by an initial burst in the first 2 h followed by sustained release up to 72 h (Figure 1f).

### 2.2 In Vitro Viability and Effect of DA‐Loaded NSs on Il‐1β and Tnf‐α Expression

**Figure 2:**
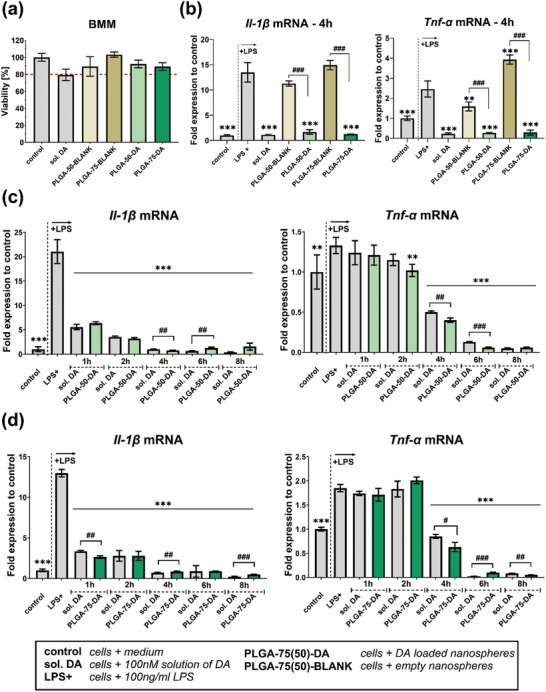
In vitro experiments with bone marrow‐derived macrophages (BMMs) treated with DA‐loaded NSs a) Viability of BMMs in the presence of DA‐loaded NSs after 24 h b) Effect of DA‐loaded NSs on mRNA expression of cytokines genes Il‐1β and Tnf‐α after 4 h c) Effect of PLGA‐50‐DA in 100 nm concentration of DA on the mRNA expression of cytokines genes Il‐1β and Tnf‐α at 1, 2, 4, 6 and 8 h compared to the effect of DA (100 nm) solution d) Effect of PLGA‐75‐DA in 100 nm concentration of DA on mRNA expression of cytokines genes Il‐1β and Tnf‐α at 1, 2, 4, 6 and 8 h compared to the effect of DA (100 nm) solution. LPS (100 ng mL^−1^) was used in the experiments to stimulate pro‐inflammatory M1 polarization (b–d). Il‐1β and Tnf‐α mRNA expression have been analyzed using RT‐qPCR with a reference gene and data are presented as fold change to untreated cells (control). ^*^ *p* < 0.05, ^**^ *p* < 0.01, ^***^ *p* < 0.001, statistically significant difference between samples and 100 ng mL^−1^ LPS‐treated control (b–d); ^#^ *p* < 0.05, ^##^ *p* < 0.01, ^###^ *p* < 0.001 statistically significant difference between empty NSs and DA‐loaded NSs (a,b) or significant difference between DA solution (100 nm) and DA‐loaded NSs using the DA concentration 100 nm (c,d). *n* = 3.

No signs of reduced viability were observed in the bone marrow‐derived macrophages (BMM) cells containing prepared formulations (**Figure** **2**a). To determine the anti‐inflammatory effects of prepared DA‐loaded NSs, BMMs were stimulated with LPS for pro‐inflammatory polarization manifested by increasing production of pro‐inflammatory cytokines Il‐1β and Tnf‐α mRNA. A significant decrease in the expression of pro‐inflammatory cytokine genes induced by both types of NSs (PLGA‐50‐DA and PLGA‐75‐DA) was observed 4 h after treatment (Figure 2b). Blank PLGA formulations without loading of DA (PLGA‐50‐BLANK, PLGA‐75‐BLANK) did not suppress the mRNA expression of pro‐inflammatory Il‐1β and Tnf‐α cytokines genes. Interestingly, PLGA‐75‐BLANK NSs display a significant (*p* < 0.05) increase in Tnf‐α mRNA expression in the presence of LPS (Figure 2b).

In the next experiments, both DA‐loaded formulations PLGA‐50‐DA and PLGA‐75‐DA induced a gradual decrease in cytokines Il‐1β and Tnf‐α mRNA expression at 1, 2, 3, 4, 6, and 8 h intervals (Figure 2c,d). The most significant suppression of Il‐1β and Tnf‐α gene mRNA expression occurred 6 and 8 h after the treatment. An equivalent concentration of DA in solution was used to compare the anti‐inflammatory effects of DA‐loaded NSs. We found no significant difference in the effects of DA‐loaded NSs and DA solution on the cytokine mRNA expression.

Based on the data, we decided to perform further experiments with PLGA 75 (PLGA‐75‐DA) NSs. DA's higher loading efficiency and PLGA‐75 hydrophobic properties should enable higher uptake by liver macrophages and better delivery of DA into cells.^[^ ^15^ ^]^

### 2.3 Confocal/Fluorescence Microscopy of fluorescently labeled NSs in the Presence of BMMs

**Figure 3:**
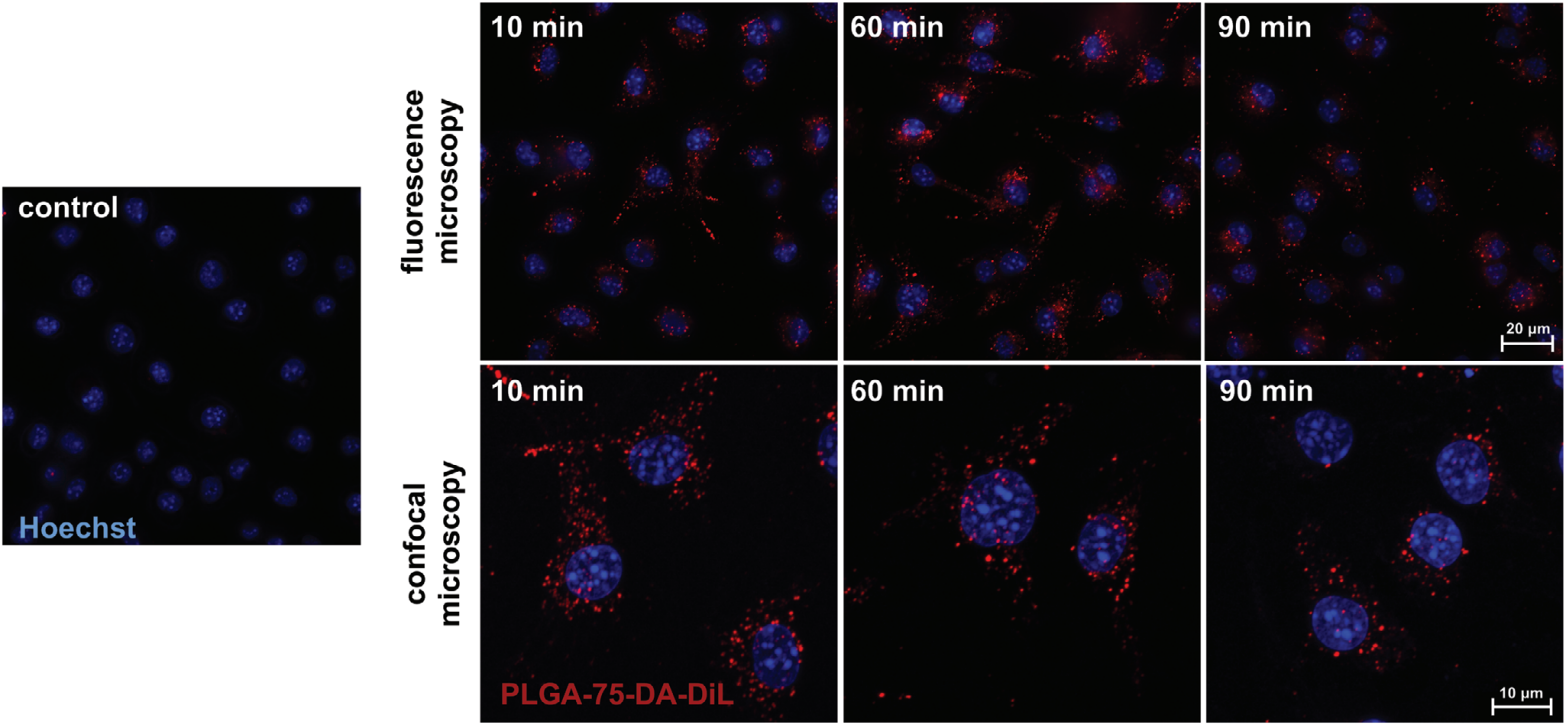
Uptake of DiL‐labeled and DA‐loaded NSs in bone marrow‐derived macrophages. Images from confocal/fluorescence microscopy after 10, 60, and 90 min; nuclei stained by Hoechst 33342 (blue color); PLGA‐75‐DA‐DiL (red color).

Fluorescent dye‐labeled NSs loaded with DA were prepared using 1,1′‐dioctadecyl‐3,3,3′,3′‐tetramethylindocarbocyanine perchlorate (DiL) dye. The uptake of PLGA‐75‐DA‐DiL by BMMs was confirmed by live‐cell imaging using fluorescence and confocal microscopy (**Figure** **3**; Video S1, Supporting information). Intracellular localization of DiL‐labeled NSs in BMMs was confirmed within 10 min after the treatment as a punctate signal throughout the cytoplasm. After 90 min NSs were still detectable in the cells. No signal was detected in non‐treated cells (control) using the same acquisition settings.

### 2.4 Flow Cytometry Analysis of the Accumulation of fluorescently labeled NSs

**Figure 4:**
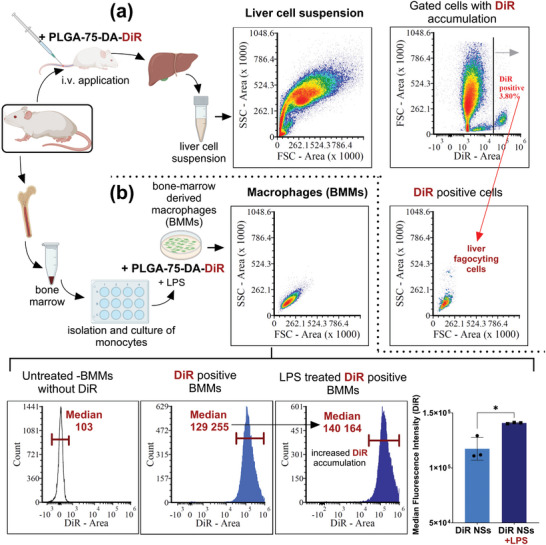
Flow cytometry analysis of single‐cell suspensions of mouse liver and bone marrow‐derived macrophages (BMMs) after treatment with DiR dye‐labeled (PLGA‐75‐DA‐DiR) NSs. a) The analysis of the single‐cell suspensions of mouse liver by flow cytometric scatter plots indicates the cell size, granularity, and viability of DiR‐positive cells. Representative flow cytometry histograms show the DiR‐labeled nanoparticles’ accumulation in the mouse liver's single‐cell suspension 1.5 h after PLGA‐75‐DA‐DiR application (0.3 mg mL^−1^ PLGA). b) Representative flow cytometry histograms showing the DiR‐labeled nanoparticles accumulation in BMMs after in vitro treatment with PLGA‐75‐DA‐DiR application (1.5 h, 0.3 mg mL^−1^ PLGA). The shift in peak area indicates increased cellular uptake after LPS treatment (100 ng mL^−1^). The cell size, granularity, and viability of BMMs correspond with the scatter plots of DiR‐positive cells of mouse liver. ^*^ *p* < 0.05 statistically significant difference between DiR positive BMMs and LPS treated DiR positive BMMs.

For the in vivo distribution experiments with mice, we prepared PLGA‐75‐DA NSs labeled with 1,1′‐dioctadecyl‐3,3,3′,3′‐tetramethylindotricarbocyanine iodide (DiR) dye, which is suitable for in vivo imaging. To follow the distribution of DiR‐labeled NSs, mice were treated i.v. with PLGA‐75‐DA‐DiR and euthanized 1.5 h after i.v. application. Subsequently, the single‐cell suspension of the liver was analyzed using flow cytometry. (**Figure** **4**a). Cells exerting the highest DiR fluorescent signal were gated, and the relative size and granularity of gated cells were determined using forward scatter cytometry (FSC) and side scatter cytometry (SSC) analysis. The position of the DiR‐positive phagocyting cells based on FSC and SSC parameters correlated well with the position of macrophages (BMMs) with accumulated PLGA‐75‐DA‐DiR. (Figure 4b) The same cell position of BMMs and DiR‐positive liver cells on scatters identify the DiR‐positive cells as liver macrophages internalizing DiR‐labeled NSs.

In addition, we examined whether LPS treatment of BMMs stimulates DiR‐labeled NSs uptake. We found that DiR fluorescence histogram intensity showed a significant shift in peak area after LPS treatment of BMMs compared to non‐LPS‐treated BMMs (Figure 4b). Thus, the analysis revealed increased uptake of DiR‐labeled nanoparticles after LPS proinflammatory stimulation of BMMs.

### 2.5 In Vivo Imaging of Specific Organ Accumulation of PLGA‐75‐DA‐DiR After i.v. Administration

**Figure 5:**
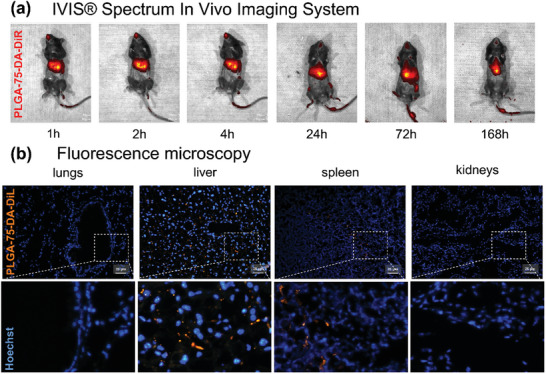
Accumulation of fluorescent DiR/DiL dye‐labeled/DA‐loaded PLGA‐75 NSs in mice after intravenous administration a) Pictures of mice obtained from IVIS Spectrum In Vivo Imaging System after i.v. application of PLGA‐75‐DA‐DiR in different time points; PLGA‐75‐DA‐DiR (red color) b) Fluorescence microscopy of tissue slides prepared 1.5 h after i.v. application of PLGA‐75‐DA‐DiL; nuclei stained by Hoechst 33342 (blue color); PLGA‐75‐DA‐DiL (orange color); scale bar 25 µm.

The DiR dye‐labeled/DA‐loaded PLGA‐75 NSs targeting the liver were also confirmed by IVIS Spectrum In Vivo Imaging System after i.v. administration in mice (**Figure** **5**a). The mice were anesthetized in 1, 2, 4, 24, 72, and 168 h intervals after the i.v. PLGA‐75‐DA‐DiR treatment, was placed into the instrument, and in vivo images were taken. We found that the accumulation of DiR‐labeled NSs was detectable after 1 h in the liver, and the signal remained for up to 168 h after treatment (Figure 5a).

For a detailed distribution view of DiL‐labeled NSs, lungs, liver, spleen, and kidneys were also harvested 1.5 h after administration and analyzed using fluorescent microscopy (Figure 5b). DiL‐labeled NSs were detectable only in the liver and spleen; no significant accumulation of NSs was observed in the lungs and kidneys.

## 3 Discussion

The PLGA NSs we prepared in the study display appropriate properties that ensure the targeting of liver macrophages.^[^ ^14^, ^15^ ^]^ The size of prepared NSs falls within the desired range of 100–300 nm.^[^ ^22^ ^]^ NSs prepared during our experiments had a size below 200 nm. Moreover, the polydispersity index below 0.2 suggests monodisperse particles with narrow size distribution (Figure 1b) and spherical morphology (Figure 1c). Such characteristics mean that prepared NSs are suitable for sterile filtration, compatible with i.v. administration, and attractive for macrophages.

Liver macrophages are capable of particle internalization via multiple receptors, including mannose, toll‐like, and Fc, via multiple mechanisms, including micropinocytosis, endocytosis, and phagocytosis.^[^ ^23^ ^]^ Particles smaller than 100 nm are not recognized by macrophages and do not act as a macrophage‐specific delivery system, as was demonstrated in our previous study.^[^ ^15^ ^]^ The sub‐100 nm particles are preferably internalized by liver‐scavenging endothelial cells. Conversely, particles larger than 300 nm cannot reach the perisinusoidal space, where the liver macrophages reside. Thus, specificity for liver macrophage delivery is dramatically reduced for particles larger than 300 nm.^[^ ^14^ ^]^ Due to the need to maintain a strict size range for effective macrophage targeting, we preferred the nanoprecipitation approach for PLGA DA NSs preparation to the widely used emulsification solvent evaporation method. The nanoprecipitation method consistently produces NSs attractive for macrophages in the desired size range.^[^ ^15^ ^]^ Additionally, it was shown that negatively charged nanoparticles are internalized at a much higher rate than neutral or positively charged ones. The zeta potential of nanoparticles prepared in this study ranged between −15 and −20 mV, thus further enhancing macrophage‐targeting properties.^[^ ^24^ ^]^

The preparation of drug‐loaded NSs was conducted with DA, a hydrophobic prodrug of well‐known GC dexamethasone. The optimal input amount of DA for NSs preparation by nanoprecipitation (600 µg DA per 1 mL of organic phase) was determined by comparing the DLE% and drug loading capacity of NSs at varying input concentrations of DA (Supplement 1, Supporting Information). The increased hydrophobicity of DA when compared to dexamethasone leads to the enhancement of DLE% up to almost 20 % (Figure 1d). Interestingly, DLE% of dexamethasone in PLGA material was reported to be less than 10%.^[^ ^18^, ^25^ ^]^ By achieving a DLE% of almost 20%, this offers a considerable advantage over the use of dexamethasone. It was demonstrated that DA shows an effectivity in low nanomolar concentrations.^[^ ^26^ ^]^ Therefore, the loaded quantity of DA should be sufficient for the anti‐inflammatory effect upon NSs reaching the target tissue. As expected, a considerably higher DLE% of DA was achieved using the more hydrophobic PLGA 75 with a higher lactic acid ratio. Based on this fact, we hypothesize that hydrophobic interactions are critical in incorporating DA into the PLGA matrix during the nanoprecipitation process.

During the in vitro experiments, no signs of toxicity were observed upon treatment of BMMs with DA‐loaded NSs (Figure 2a; Supplement 2, Supporting Information). The confocal/fluorescence microscopy confirmed the intracellular localization of traceable PLGA‐75‐DA‐DiL NSs. The immediate uptake of PLGA‐75‐DA‐DiL by BMMs in less than 10 min showed the expected attractiveness of the PLGA NSs for macrophages (Figure 3). Consequent PCR experiments showed that loading of DA into PLGA NSs does not interfere with the capability of DA to suppress the production of pro‐inflammatory cytokines under equimolar concentrations of 100 nm (Figure 2c,d). For a detailed dose‐response experiment, please see Supplement 3 (Supporting Information). In addition, our drug release data show sustained release of DA for 72 h (Figure 1f). Therefore, we hypothesize that DA‐loaded NSs can effectively suppress inflammation for a period of up to 72 h. Moreover, dexamethasone is a long‐acting corticosteroid with a biological half‐life of 36–72 h.^[^ ^27^ ^]^ As DA is a lipophilic prodrug of dexamethasone, its biological half‐life is longer than that of dexamethasone. Importantly, sustained release from PLGA NSs presents a substantial advantage over conventional daily dexamethasone administration, significantly prolonging DA biological effectivity.

DA loading into PLGA also leads to the specificity of distribution. We demonstrate that after i.v. administration to mice, the PLGA NSs are specifically internalized by liver macrophages. Other non‐target cells, such as hepatocytes and hepatic stellate cells, did not show any accumulation of PLGA‐75‐DA‐DiR NSs (Figure 4a). Alteration of metabolic pathways in hepatocytes is the primary cause of dexamethasone side effects. Our findings suggest that the administration of DA‐loaded NSs could translate into a situation where minimal or no side effects are triggered as DA is not delivered into non‐target cells such as hepatocytes. Moreover, the specificity of distribution means that the DA dose sufficient for effective suppression of liver inflammation will be considerably lower than the conventional administration since the ubiquitous distribution into the whole body will be minimized. It was also demonstrated by the flow cytometry analysis of isolated BMMs with DiR‐labeled NSs that PLGA NSs were preferably accumulated in LPS‐induced M1 pro‐inflammatory macrophages (Figure 4b).^[^ ^28^ ^]^ M1 macrophage polarization may provide a possible explanation for this by increasing phagocytic activity.^[^ ^29^ ^]^

The flow cytometry data demonstrate that macrophages can internalize PLGA NSs while other cell populations are not targeted. These findings were further supported by organ distribution experiments, which show the presence of PLGA‐75‐DA‐DiL in macrophage‐rich organs such as the liver and spleen. DiL‐labeled NSs were visualized within liver sinusoids, the site of Kupffer cells localization, and in the marginal zone of the spleen, the area with a complex macrophage network (Figure 5b).^[^ ^30^ ^]^ The absence of a DiL dye signal in the systemic circulation suggests that PLGA‐75‐DA‐DiR NSs is a macrophage‐specific carrier system (Figure 5a), unlike previously published studies with dexamethasone‐loaded liposomes that were internalized by circulating monocytic cells.^[^ ^31^ ^]^ The lack of fluorescence in the kidney suggests that no free dye was released from PLGA‐75‐DA‐DiL, therefore, no carrier disintegration occurred (Figure 5a,b).

Our study presents biodegradable polymeric NSs as an effective macrophage‐specific delivery system for DA. Although promising in vitro and in vivo results were obtained, we are aware of limitations that may appear in future phases of the project. Further research will focus on the efficacy of DA‐loaded NSs in murine models of acute liver inflammation. The primary challenge will be to deliver sufficient DA via PLGA NSs to induce anti‐inflammatory effects in vivo.

## 4 Conclusion

This study demonstrates the potential of biodegradable PLGA NSs as a targeted drug delivery system for GCs, outlining a promising approach to treating liver inflammation. Liver targeting was achieved by tuning NSs size, surface charge, and hydrophobicity of a PLGA polymer to enhance NSs uptake by liver macrophages. By delivering GCs specifically to macrophages, these nanoformulations have the potential to reduce the dose required to reach the hepatic tissue, providing a more effective and safer therapy. The prepared DA‐loaded NSs showed significant efficacy in suppressing pro‐inflammatory cytokines, specifically Tnf‐α and Il‐1b mRNA expression, in BMMs. Additionally, following i.v. administration in mice, these formulations demonstrated selective accumulation within liver macrophages while other cell populations, such as hepatocytes, which are the site of undesirable side effects, were not targeted. These findings highlight the potential of PLGA‐based nanocarriers for targeted GC therapy of hepatic inflammation. Future studies, including ongoing in vivo proof‐of‐concept experiments in liver inflammatory murine models, are essential to validate these results and further optimize this nanocarrier system.

## 5 Experimental Section

### Materials

PLGA polymer PURASORB 5002 (PLGA 50, LA:GA–50:50, ester termination, Mw = 17000 g mol^−1^) and PURASORB 7502A (PLGA 75, LA:GA–75:25, acid termination, Mw = 17000 g mol^−1^) were purchased from Corbion (Amsterdam, Netherlands). Dexamethasone 21‐acetate (DA), Pluronic F‐127, acetone, TRI Reagent, LPS from *Escherichia coli* O111:B4 (LPS), fetal bovine serum (FBS), phosphate‐buffered saline (PBS) were purchased from Merck (Darmstadt, Germany). Acetonitrile HPLC grade (purity >99.9%) was purchased from Honeywell (Charlotte, NC, USA). Dulbecco's Modified Eagle Medium (DMEM), RevertAid RT Kit, TaqMan Fast Advanced Master mix, TaqMan assays for *Il‐1β* (Mm00434228_m1), *Tnf‐α* (Mm00443258_m1), glyceraldehyde‐3‐phosphate dehydrogenase *(Gapdh)* (Mm99999915_g1), beta‐2‐microglobulin *(B2* *m)* (Mm00437762_m1), 1,1′‐dioctadecyl‐3,3,3′,3′‐tetramethylindocarbocyanine perchlorate (DiL), 1,1′‐dioctadecyl‐3,3,3′,3′‐tetramethylindotricarbocyanine iodide (DiR), Hoechst 33342 Solution, penicillin‐streptomycin solution (10000 U ml^−1^) were purchased from ThermoFisher Scientific (Waltham, MA, USA), CellTiter 96 Aqueous One Solution Cell Proliferation Assay was purchased from Promega, (Madison, WI, USA).

### PLGA Nanospheres Preparation and Purification

The nanoprecipitation method was used for NSs preparation.^[^ ^15^ ^]^ Briefly, 30 mg PLGA and 600 µg DA were dissolved in 1 mL of acetone and injected into 10 mL of 0.1% (w/v) aqueous Pluronic F127 solution under constant magnetic stirring (300 rpm). Traceable NSs were labeled by adding 30 µg of DiR (emission maximum 773 nm suitable for in vivo imaging) or DiL (emission maximum 565 nm suitable for in vitro imaging) dyes into the organic phase. The mixture was stirred for 2 h to ensure complete acetone evaporation. Evaporated water was replaced with deionized (DI) water for a total nanosuspension volume of 10 mL. Nanosuspension was consequently filtered through a 1.2 µm filter (ReliaPrepTM, 1,2 CA, Ahlstrom Munksjö, Helsinki, Finland) to remove DA crystals. Samples for in vitro and in vivo experiments were further filtered under aseptic conditions through a 0.45 µm sterile filter (Minisart, cellulose acetate membrane, 28 mm, Sartorius, Gottingen, Germany). Filtered suspension of NSs was then centrifugated at 8000 × g for 15 min at 14 °C. The supernatant was removed and centrifugated at 10 000 × g for 15 min at 14 °C. Pelleted NPs from both centrifugations were re‐dispersed in 1 mL of 5% (w/v) sterile trehalose solution and merged under aseptic conditions. Aliquots of NSs in 5% (w/v) sterile trehalose solution were subsequently stored at −70 °C.

### PLGA Nanospheres Characterization

Prepared NSs were characterized in terms of their size, polydispersity, and zeta potential using dynamic light scattering (Zetasizer Nano ZS, Malvern Panalytical, Malvern, UK). Samples were diluted with DI water to a final PLGA concentration of 1.5 mg mL^−1^ and placed into a standard polystyrene cuvette. The intensity of the scattered light was detected at a backscattering angle of 173°. The size and polydispersity of NSs were calculated based on the intensity size distribution protocol. This setup was chosen to avoid biased results by varying the optical properties of fluorescently labeled NSs. The viscosity of media was selected to that of the DI water. All samples were measured three times, each involving at least 12 independent runs. Zeta potential was determined using electrophoretic light scattering. For zeta‐potential measurement, the attenuator was set to automatic mode. DTS1070 folded capillary cuvettes were used.

Scanning electron microscopy (SEM) (HITACHI FlexSEM 1000, Hitachi, Tokyo, Japan) was used to confirm the size and morphology of NSs. Prepared samples were diluted with DI water at a ratio of 1:50. The droplet of the sample was applied to a microscope slide and dried at room temperature (RT). The sample was sputtered with 8 nm of platinum to increase the surface conductivity. The sample was observed in a high vacuum using secondary electrons. An accelerating voltage of 11 kV proved to be the most advantageous.

A high‐performance liquid chromatography (HPLC) technique was used to determine the DLE%. Two milliliters of nanosuspension in sterile water were centrifugated at 15100 × g for 15 min at 14 °C. The supernatant was discarded, and pelleted NSs were dissolved in 1 mL of acetonitrile and analyzed by HPLC. Agilent Technologies 1260 Infinity (Santa Clara, CA, USA), precolumn (ARION Guard System holder for 5 mm cartridges) + (ARION 5 mm cartridges for Guard System, RP 5.0 µm, ID 4.0 mm) (ARION, Heerlen, Netherlands), column Realtek C18 were used. As the mobile phase 50% of acetonitrile and 50% of MilliQ water with a flow rate of 1 mL min^−1^ were used. The injection volume of the sample was 10 µL. DA was detected at 237 nm in retention time cca 5.4 min. in RT. DLE was calculated by referring to the amount of loaded DA and the total amount of used DA to prepare NSs.

### Drug Release Assay

The adopted method^[^ ^32^ ^]^ was used to determine DA release from NSs. Two ml tubes with a removed center of a lid (Protocol, Supporting information) were prepared and filled with NSs suspension. A piece of cellulose membrane (molecular weight cut‐off 14 kDa) was placed to cover the lid. Samples were placed into the beaker with 1200 mL of PBS and incubated at 37 °C on a shaker. Samples were drawn at predetermined intervals followed by centrifugation at 15 100 × g at 14 °C for 15 min. The amount of DA remaining within NSs was determined using HPLC (see Section 2.3).

### Macrophage Isolation, Differentiation, and Exposure

Bone marrow‐derived macrophages (BMMs) were derived from 6 to 10‐week‐old BALB/c female mice (Velaz, Prague, Czech Republic), as described by Weischenfeldt and Porse,^[^ ^33^ ^]^ with minor modifications. Briefly, the bone marrow cells were flushed out from dissected femurs and tibias. The obtained cells were differentiated into macrophages in 100 mm bacterial Petri dishes in DMEM, supplemented with the heat‐inactivated 10% (v/v) FBS, 20% L929‐conditioned medium prepared in our lab (source of macrophage‐colony stimulating factor), and antibiotics—50 µg mL^−1^ streptomycin and 50 U mL^−1^ penicillin (for the first 3 days of cultivation only) at 37 °C and 5% CO_2_. On day 7, the differentiated cells were detached by incubation in ice‐cold PBS (4 °C, up to 10 min) followed by gentle pipetting. Pelleted cells were resuspended in fresh DMEM with 10% FBS and seeded onto 48 or 96‐well polystyrene plates at 250 000 cells cm^−2^ density. All experiments on mice were conducted under the supervision of the institution's Animal Unit and were approved by the Animal Care and Use Committee of the Military Faculty of Medicine, University of Defense, Hradec Kralove, Czech Republic under project number 5/21. The study was conducted in accordance with the local legislation and institutional requirements.

To evaluate the anti‐inflammatory effect of the prepared nanoformulations, the BMMs were treated with LPS at a 100 ng mL^−1^ concentration for 2 h for M1 polarization.^[^ ^34^ ^]^ A solution of DA at 100 nm concentration was used as a standard for the GC effect in the experiments, validating the method. This concentration was selected based on published data, as it is considered a standard for inhibition of the production of inflammatory cytokines.^[^ ^35^, ^36^ ^]^ The cells were treated with DA NSs using the same 100 nm concentration of DA, according to their DLE%. Pro‐inflammatory activity in BMMs was evaluated by the analysis of pro‐inflammatory cytokines, *Il‐1β*, and *Tnf‐α* mRNA expression, using RT‐qPCR at 1, 2, 4, 6, and 8 h after treatment.

### Cell Viability Assay

The MTS assay (CellTiter 96 Aqueous One Solution Cell Proliferation Assay) was used per the manufacturer's protocol for testing of BMMs viability. Briefly, 24 h after cell seeding, NSs were added. After 24 h, the cells were twice washed with PBS and incubated with the CellTiter reagent for 1 h at 37 °C. After that, absorbance at 490 nm was measured, and cell viability was determined relative to vehicle (sterile water)‐treated cells (100% viability). A 10% (w/v) sodium dodecyl sulfate solution was used as the cytotoxic control (0% viability). The experiment was repeated three times (*n* = 3), and the tested formulations were evaluated in triplicates in all experiments. The threshold of 80% viability was used as the limit for potential cytotoxicity.

### Real‐Time Quantitative PCR (RT‐qPCR)

RNA isolation was performed using TRI Reagent according to the manufacturer's protocol. EconoSpin columns (Epoch Life Science, Missouri City, TX, USA) were used for purification. The purity and the concentration of RNA were measured using a NanoDrop spectrophotometer (ThermoFisher Scientific, Waltham, MA, USA). For the transcription, the RevertAid RT Kit was used. The qRT‐PCR experiments were performed using the QuantStudio 6 Real‐Time PCR System with TaqMan Fast Advanced Master mix. Pro‐inflammatory activity in BMMs was evaluated by the analysis of two cytokines, *Il‐1β*, and *Tnf‐α* mRNA expression, using commercial TaqMan assays. The housekeeping *Gapdh* and *B2* *m* genes were used as internal standards. PCR reactions were performed using technical replicates. The delta‐delta method was used for relative mRNA expression quantification. Data are presented as fold change to control samples (untreated samples). RT‐qPCR experiments were performed in triplicates, and the experiments were repeated three times (*n* = 3).

### Confocal/Fluorescence Microscopy

For confocal microscopy, BMMs were seeded onto confocal dishes (µ‐Dish 35 mm, high Glass Bottom, Ibidi GmbH, Gräfelfing, Germany) at a 100 000 cells cm^−2^ density. After overnight stabilization, cells were treated with vehicle (DMEM) for control or DiL‐labeled NSs at a concentration of 0.03 mg mL^−1^ PLGA. After 10, 60, and 90 min medium was aspirated, the cells were washed twice with PBS, and the new medium with Hoechst 33342 (1 ng mL^−1^, 5 min at 37 °C) was used for nuclear staining of living cells. Image acquisition was performed using Nikon A1+ laser scanning confocal system (based on Nikon Ti‐E microscope; Nikon Instruments, Tokyo, Japan), NIS Elements AR 4.2 acquisition software, Nikon Plan Apo λ 100× Oil immersion objective lens, and, 405 and 561 nm lasers for DAPI and Cy3.5 channels, respectively. All images were taken with 512 × 512 pixel resolution with 2.1 confocal zoom (giving 0.12 µm pixel size). Each image is a maximum intensity projection stack of 38–64 focal planes (to cover the whole volume of each sample; 150 nm z‐step). The pinhole was set at 57.5 µm and dwell time at 1.9 µs. Prior to confocal microscopy, samples were checked with fluorescence microscopy using the same instrument equipped with Andor Zyla 5.5 sCMOS cooled camera (Andor Technology Ltd, Oxford Instruments plc, Abingdon, UK), CoolLED pE‐300 (CoolLED Limited, Andover, UK) fluorescence source using DAPI and Cy3 filter sets.

### Animals

Male C57BL/6J mice (Charles River Laboratories, Cologne, Germany) were housed in a temperature‐ and humidity‐controlled room with a 12 h alternating light‐dark cycle period. Mice were fed a standard pelleted diet (Altromin, Lage, Germany) and freshwater ad libitum. The care and handling of mice conformed to European Union recommendations on handling experimental animals and was approved by the Animal Welfare Body of Charles University (Hradec Kralove, Czech Republic) project no. MZE‐47683/2021‐18134.

### Flow Analysis

For flow cytometry analysis, BMMs were seeded as described (see Section 2.5) and treated with DiR‐labeled NSs at a 0.3 mg mL^−1^ PLGA concentration. M1 polarization by LPS was performed. After 1.5 h, the treatment was stopped by washing the cells twice with ice‐cold PBS. The cell viability was determined using the LIVE/DEAD Fixable Scarlet viability kit (ThermoFisher Scientific, Waltham, MA, USA). The uptake of DiR‐labeled NSs was analyzed by flow cytometry using the SA3800 spectral analyzer (Sony Biotechnology, San Jose, CA, USA).

C57BL/6J mice were treated i.v. with DiR‐labeled NSs at a concentration of 0.3 mg mL^−1^. The injection volume was 100 µL. After 1.5 h, mouse liver cells were isolated by a retrograde two‐step collagenase perfusion method. A fraction rich in Kupffer cells and other nonparenchymal liver cells was collected from the upper part of the suspension of isolated cells. The cell viability was determined using the LIVE/DEAD Fixable Scarlet viability kit. The uptake of DiR‐labeled NSs was analyzed by flow cytometry using the SA3800 spectral analyzer.

### In Vivo Imaging

C57BL/6J mice were anesthetized by inhalation of 2% isoflurane and trimmed with a hair clipper in the region of interest. Mice were treated with DiR‐labeled and DA‐loaded PLGA‐75 NSs by i.v. injection (volume 100 µL) at a concentration of 0.3 mg mL^−1^ PLGA. At predetermined time intervals (1, 2, 4, 24, 72, and 168 h), in vivo imaging analysis using IVIS Spectrum (Perkin Elmer Inc., Waltham, MA, USA) was performed to visualize the distribution of DiR‐labeled NSs. Mice were anesthetized using 2% isoflurane and placed into the imaging chamber. The system was set to acquire images with a 745 nm excitation filter and an 800 nm emission filter for fluorescence imaging. Images were analyzed using Living Image 4.4 software.

### Tissue Slides

C57BL/6J mice were treated i.v. with DiL‐labeled NSs at a concentration of 0.3 mg mL^−1^ PLGA. After 1 h, mice were euthanized, and the liver, spleen, kidneys, and lungs were fixed with 4% paraformaldehyde, embedded in OCT cryoprotective medium (Leica, Wetzlar, Germany), and 5 µm cryosections were cut. Nuclei were counterstained with Hoechst 33342, and fluorescence images were captured using Olympus AX 70 microscope with an incorporated Nikon DS‐Fi3 high‐definition color microscope camera and image analysis software NIS (Laboratory Imaging, Praha, Czech Republic).

### Statistical Analysis

The Prism 10 program (GraphPad, San Diego, CA, USA) was used to statistically analyze RT‐qPCR and cytotoxicity assays. The statistical significance of differences between the means of individual groups was calculated using a one‐way analysis of variance (ANOVA) with Dunnett's post hoc test. Additionally, we used a student's unpaired *t*‐test to compare the two groups of values. A *p*‐value less than 0.05 (*p* < 0.05) was considered statistically significant.

## Conflict of Interest

The authors declare that no conflict of interest.

## Supporting information



Supporting Information

Supporting Information

Supplemental Movie 1

Supplemental Movie 2

Supplemental Movie 3

## Data Availability

The data that support the findings of this study are available from the corresponding author upon reasonable request.
