# Supplementary material for: Dexamethasone Acetate‐Loaded PLGA Nanospheres Targeting Liver Macrophages
Source: Macromol Biosci. 2024 Nov 29;25(2):2400411. doi: 10.1002/mabi.202400411 (PMC11827543; doi:10.1002/mabi.202400411)
Supplement: Supplementary file 1 — Supporting Information [file MABI-25-2400411-s005.docx]

Supporting Information

**Dexamethasone Acetate-Loaded PLGA Nanospheres Targeting Liver Macrophages**

*Barbora Boltnarova, Anna Durinova, Lenka Jandova, Stanislav Micuda, Otto Kucera, Ivona Pavková, Miloslav Machacek, Ivana Nemecková, Marek Vojta, Jan Dusek, Maria Krutakova, Petr Nachtigal, Petr Pavek, Ondrej Holas**

Method:

**Drug loading efficacy (DLE)** was determined by HPLC. Two milliliters of nanosuspension in sterile water was centrifugated at 15,100 × g for 15 min at 14°C. The supernatant was discarded, and pelleted NSs were dissolved in 1 ml of acetonitrile and analyzed by HPLC. Agilent Technologies 1260 Infinity (Santa Clara, CA, USA), precolumn (ARION® Guard System holder for 5 mm cartridges) + (ARION® 5 mm cartridges for Guard System, RP 5.0 μm, ID 4.0 mm) (ARION, Heerlen, Netherlands), column Realtek C18 were used. As the mobile phase 50 % of acetonitrile and 50 % of MilliQ water with a flow rate of 1 ml min^-1^ were used. The injection volume of the sample was 10 μL. Dexamethasone acetate (DA) was detected at 237 nm in retention time cca 5.4 min. in RT. DLE was calculated by referring to the amount of loaded DA and the total amount of used DA to prepare nanospheres (NSs).

**Drug loading capacity (DLC)** was calculated as the ratio between the weight of loaded DA and the weight of the formulation. The amount of DA was analyzed by HPLC using the same protocol as DLE. The weight of the formulation was determined gravimetrically. The nanosuspension was centrifugated at 15,100 × g for 15 min at 14°C. After removing the supernatant, the pellet was dissolved in acetonitrile and transferred into a calibrated petri dish. The acetonitrile was gently evaporated at room temperature in a fume hood, and the formulation was weighted.

Both DLE and DLC were evaluated for different input concentrations of DA: 0.5, 1, 2, 5, 10% (0.15, 0.3, 0.6, 1.5, 3 mg ml^-1^ of DA). Experiments were performed in triplicate (*n* = 3).


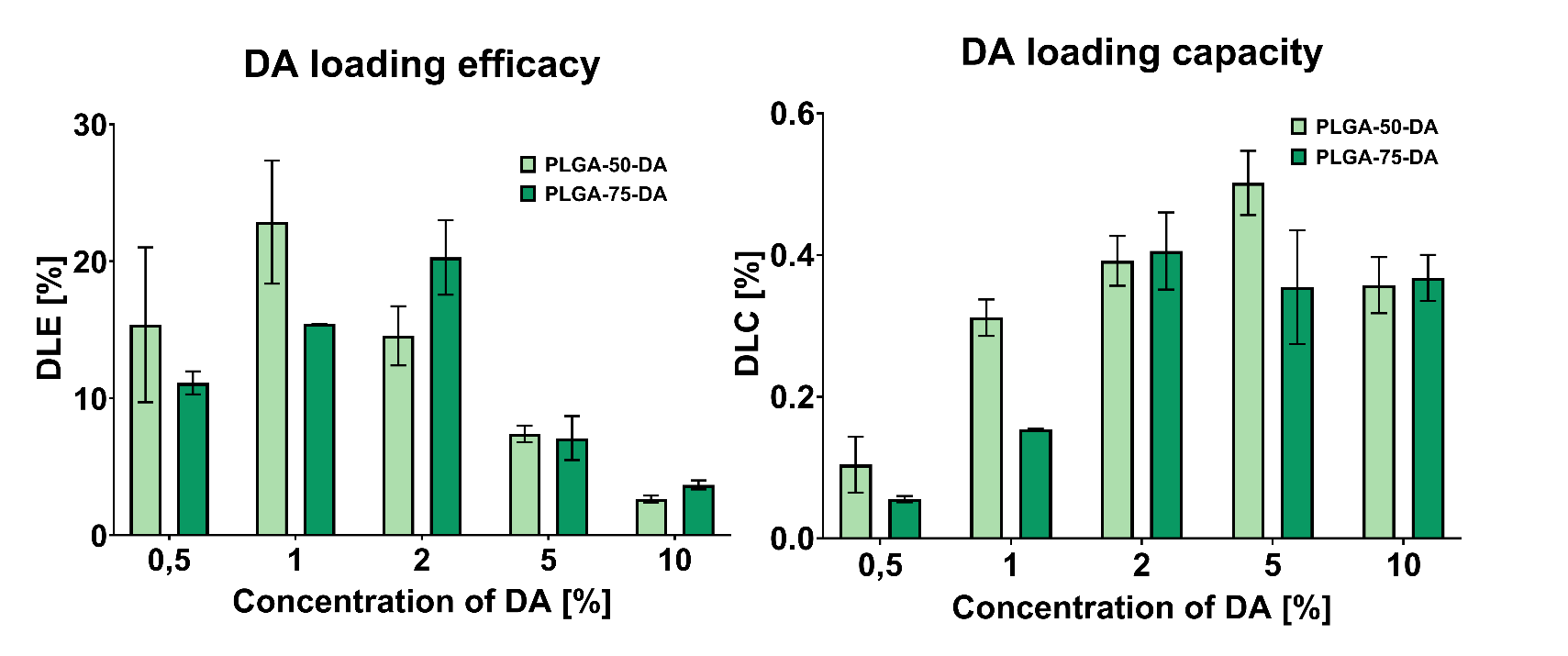


Supplement 1: Dexamethasone acetate (DA) loading efficacy (DLE) and loading capacity (DLC) of prepared PLGA NSs using PLGA 50 and PLGA 75 at varying input concentrations of DA: 0.5, 1, 2, 5, 10% (0.15, 0.3, 0.6, 1.5, 3 mg ml^-1^ of DA). *n* = 3

Method:

**Drug concentration-dependent cell viability assay**

Bone marrow-derived macrophages (BMMs) were derived from 6–10-week-old BALB/c female mice (Velaz, Praha, Czech Republic), as described by Weischenfeldt and Porse, with minor modifications. Briefly, the bone marrow cells were flushed out from dissected femurs and tibias. The obtained cells were differentiated into macrophages in 100mm bacterial Petri dishes in DMEM, supplemented with the heat-inactivated 10% (v/v) FBS, 20% L929-conditioned medium prepared in our lab (source of macrophage-colony stimulating factor), and antibiotics—50 µg ml^‑1^ streptomycin and 50 U ml^-1^ penicillin (for the first three days of cultivation only) at 37°C and 5% CO_2_. On day 7, the differentiated cells were detached by incubation in ice-cold PBS (4 °C, up to 10 min) followed by gentle pipetting. Pelleted cells were resuspended in fresh DMEM with 10% FBS and seeded onto 48 or 96-well polystyrene plates at 250,000 cells cm^-2^ density. All experiments on mice were conducted under the supervision of the institution’s Animal Unit and were approved by the Animal Care and Use Committee of the Military Faculty of Medicine, University of Defense, Hradec Kralove, Czech Republic under project number 5/21. The study was conducted in accordance with the local legislation and institutional requirements.

The MTS assay (CellTiter 96^®^ Aqueous One Solution Cell Proliferation Assay) was used per the manufacturer’s protocol for viability testing of BMMs. Briefly, 24 h after cell seeding, samples were added. PLGA-75-DA and PLGA-50-DA were added at DA concentrations of 100 nm, 10 nm, 1 nm. Additionally, a DA solution was added at equivalent concentrations 100 nm, 10 nm, 1 nm. Samples without DA loading (PLGA-50-BLANK, PLGA-75-BLANK) were used in quantities corresponding to DA-loaded samples (PLGA-50-DA, PLGA-75-DA). After 24 h, the cells were twice washed with PBS and incubated with the CellTiter reagent for one hour at 37°C. After that, absorbance at 490 nm was measured, and cell viability was determined relative to vehicle (sterile water)-treated cells (100% viability). A 10% (w/v) sodium dodecyl sulfate (SDS) solution was used as the cytotoxic control (0% viability). The experiment has been repeated three times (n=3), and the tested formulations were evaluated in triplicates in all experiments. The threshold of 80% viability was used as the limit for potential cytotoxicity.


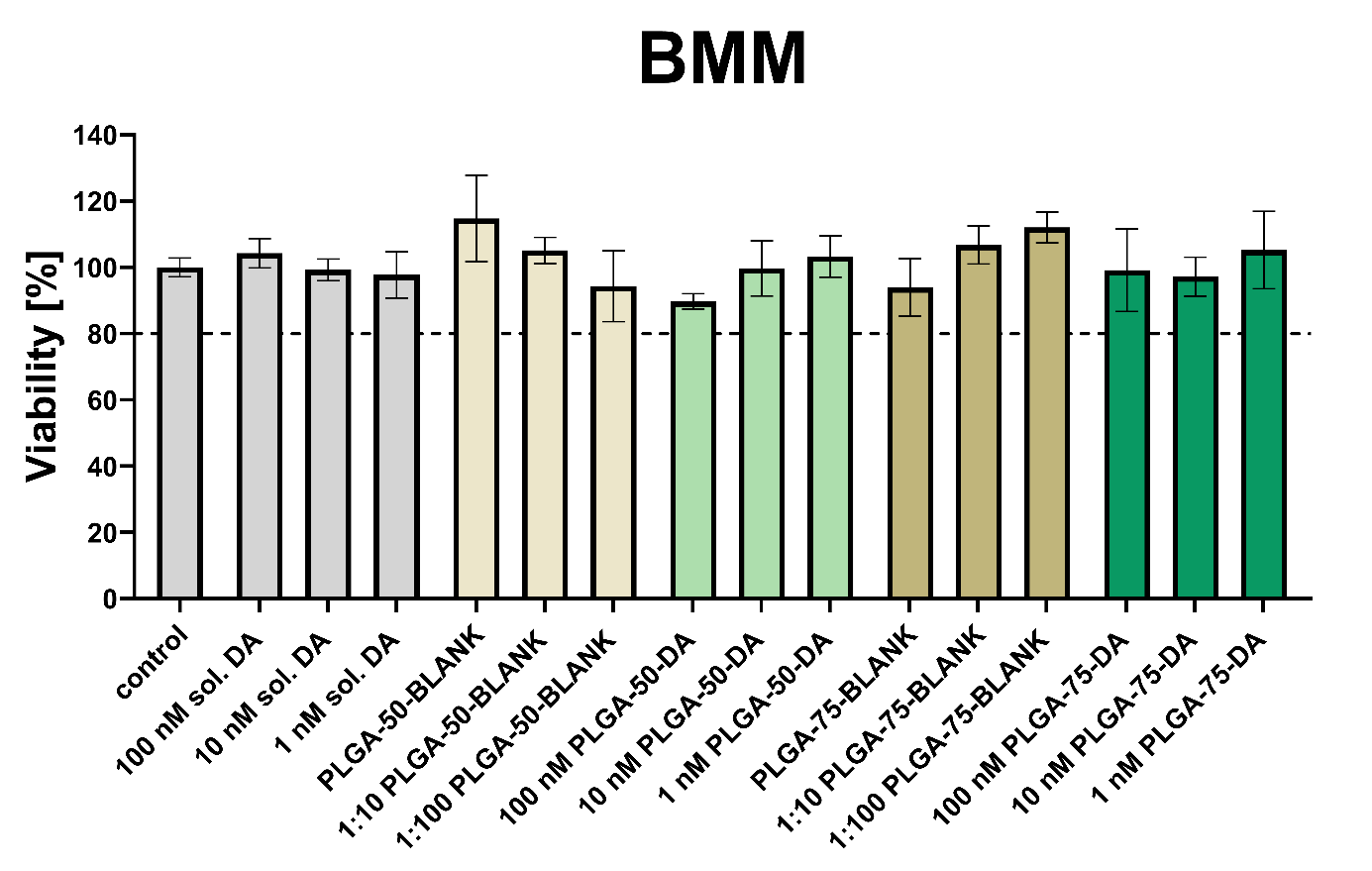


Supplement 2: Drug concentration-dependent cell viability assay in bone marrow-derived macrophages (BMM) 24 h after treatment of prepared PLGA NSs (PLGA-75-DA and PLGA-50-DA) at DA concentrations of 100 nm, 10 nm, 1 nm and DA solution at equivalent concentrations 100 nm, 10 nm, 1 nm using MTS assay. Samples without DA loading (PLGA-50-BLANK, PLGA-75-BLANK) were used in amounts corresponding to DA-loaded samples (PLGA-50-DA, PLGA-75-DA). The threshold of 80% viability was used as the limit for potential cytotoxicity. *n* = 3.

Method:

**Dose response assay**

The mouse monocyte macrophage cell line J774.2 (purchased from Sigma-Aldrich, ECACC ref No: 85011428, Saint Louis, MO, USA) was cultured in Dulbecco’s Modified Eagle Medium (DMEM, Sigma-Aldrich, Saint Louis, MO, USA) supplemented with 10% of fetal bovine serum (FBS, Sigma-Aldrich, Saint Louis, MO, USA) at 37°C in the presence of 5% CO2. Cells were seeded onto 24-well polystyrene plates at 200,000 cells cm-2 density. Macrophages were treated with LPS at a concentration of 100 ng ml‑1 for two hours to induce M1 polarization. A solution of DA and both nanoformulations, PLGA-50-DA and PLGA-75-DA, were added to the cells at DA concentrations of 0.1, 1, 10, 100 and 300 nm. After 6 h, Il-1β mRNA expression was evaluated using RT-qPCR, and inhibition curves for the DA solution, PLGA-50-DA and PLGA-75 were analyzed.

RNA isolation was performed using TRI Reagent® according to the manufacturer’s protocol. EconoSpin® columns (Epoch Life Science, Missouri City, TX, USA) were used for purification. The purity and the concentration of RNA were measured using a NanoDrop spectrophotometer (ThermoFisher Scientific, Waltham, MA, USA). For the transcription, the RevertAid RT Kit was used. The qRT-PCR experiments were performed using the QuantStudio 6 Real-Time PCR System with TaqMan Fast Advanced Master mix. Pro-inflammatory activity in BMMs was evaluated by the analysis of cytokine Il-1β mRNA expression, using commercial TaqMan assays. The housekeeping glyceraldehyde-3-phosphate dehydrogenase (Gapdh) and beta-2-microglobulin (B2m) genes were used as internal standards. PCR reactions were performed using technical replicates. The delta-delta method was used for relative mRNA expression quantification. Data are presented as fold change to control samples (untreated samples). RT-qPCR experiments were performed in triplicates, and the experiments were repeated three times (n=3).


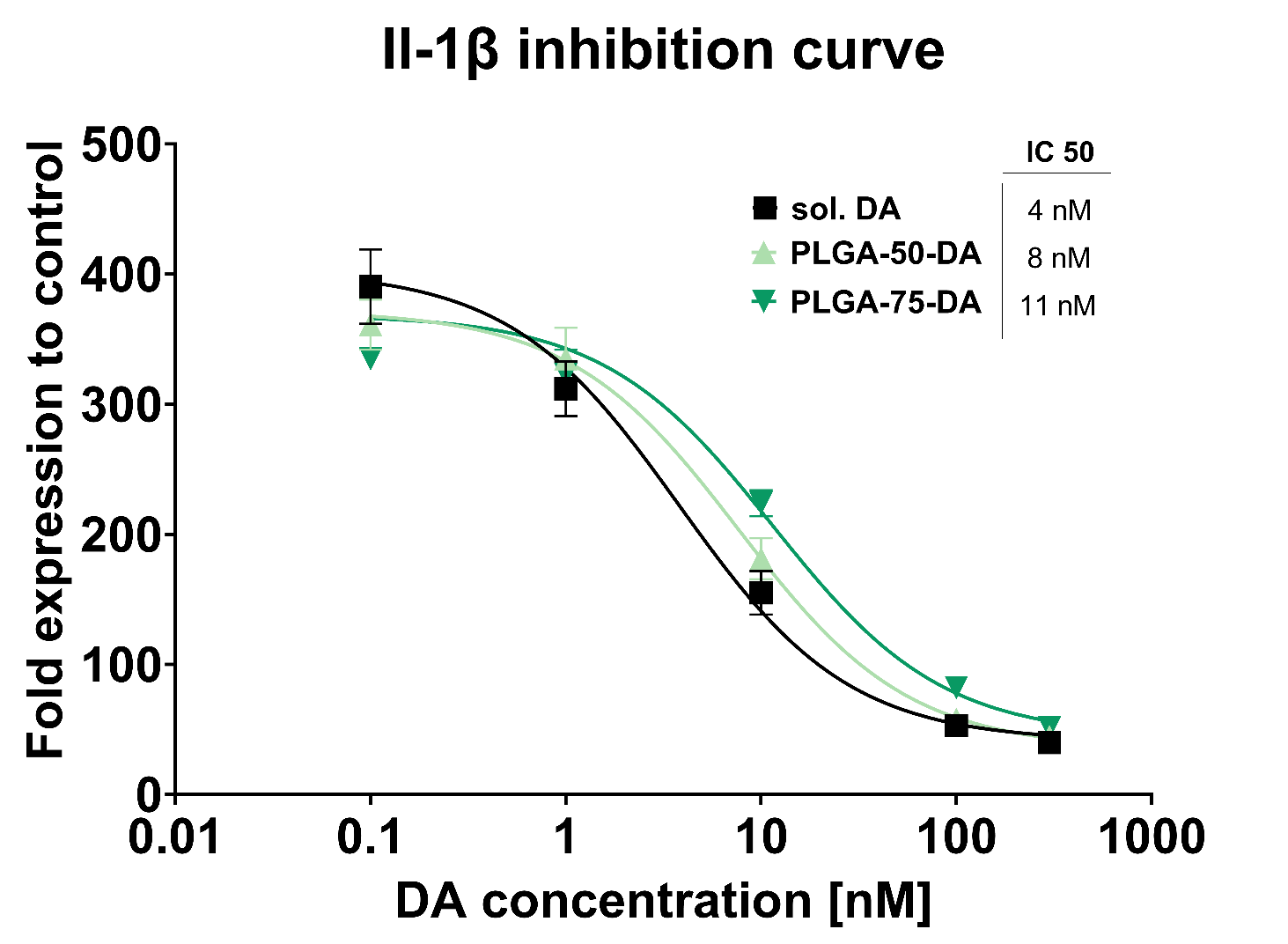


**Supplement 3:** Dose response assay – Il-1β inhibition curves of DA solution and prepared nanoformulations (PLGA-50-DA and PLGA-75-DA) in J774.2 mouse macrophage cell line. A solution of DA and both nanoformulations (PLGA-50-DA and PLGA-75-DA) were added to the cells at DA concentrations of 0.1, 1, 10, 100 and 300 nm. Macrophages were treated with LPS at a concentration of 100 ng ml^-1^ to induce M1 polarization. Inhibition curves were generated by evaluating Il-1β mRNA gene expression using RT-qPCR 6 h after treatment. *n* = 3

Received: ((will be filled in by the editorial staff))
Revised: ((will be filled in by the editorial staff))
Published online: ((will be filled in by the editorial staff))
