# Supplementary material for: Dexamethasone Acetate‐Loaded PLGA Nanospheres Targeting Liver Macrophages
Source: Macromol Biosci. 2024 Nov 29;25(2):2400411. doi: 10.1002/mabi.202400411 (PMC11827543; doi:10.1002/mabi.202400411)

Macromolecular Bioscience

Supporting Information

for *Macromol. Biosci.*, DOI 10.1002/mabi.202400411

Dexamethasone Acetate-Loaded PLGA Nanospheres Targeting Liver Macrophages

*Barbora Boltnarova, Anna Durinova, Lenka Jandova, Stanislav Micuda, Otto Kucera, Ivona Pavkova, Miloslav Machacek, Ivana Nemeckova, Marek Vojta, Jan Dusek, Maria Krutakova, Petr Nachtigal, Petr Pavek and Ondrej Holas**

Drug release assay

For determination of DA release from NSs we used method with 2 ml tubes with removed centre of lid, the method was modified based on the protocol by Andrew et al., 1999 (**Figure 1**). This procedure was chosen because we struggled with analysing of small concentrations of released DA through standard dissolution tests. Therefore, the opposite approach was chosen, not the release of DA but the decrease of DLE% of our NSs was observed, which is easier to analyse.

NSs with DA were prepared as mentioned in paragraph NSs preparation (2.2.). After filtration through 1.22 um filter suspension was divided into 2 ml tubes with drilled top where is placed piece of membrane (cut-off 14 kDa). Samples were placed in 3 l beaker with 1200 ml of media, incubated at 37 °C on a shaker. At predetermined time intervals samples were drawn followed by centrifugation at 15100 x g at 14 °C for 15 min. The protocol for determination of DLE% was used (2.3.). The decrease of loaded DA in NSs was determined and the drug release curve was calculated.

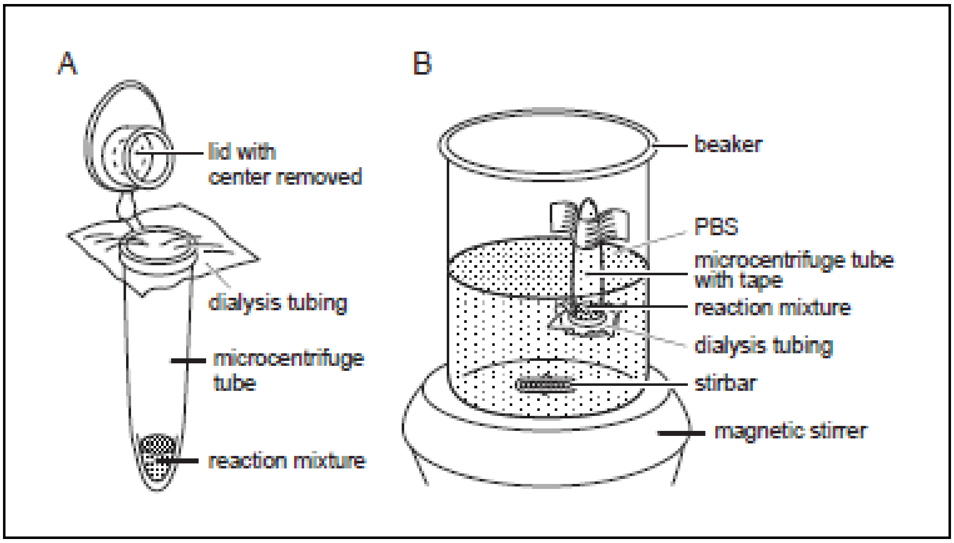

Figure A.3C.1 Making a microdialysis chamber. (A) Remove the center of the cap of a microcentrifuge tube with a heated Pasteur pipet (wide end). Place reaction mixture to be dialyzed into the tube. Place a piece of softened dialysis membrane loosely over top of tube. (B) Invert tube, shake sample down onto dialysis membrane (or centrifuge the tube inverted, if the sample is small), and tape tube into a beaker of buffer. Check that no bubbles are caught under the membrane.

Figure 1. Schematic diagram of drug release assay published in Sarah.M. Andrew, Julie.A. Titus, L. Zumstein, Dialysis and Concentration of Protein Solutions, Curr Protoc Cell Biol 4 (1999). https://doi.org/10.1002/0471143030.cba03cs04.

Photodocumentation of the procedure

1. Remove lid of 2 ml epp tube. Add small piece of dialysis membrane fitted inside lid. Fill epp with 2 ml of nanoparticle suspension.

dialysis membrane

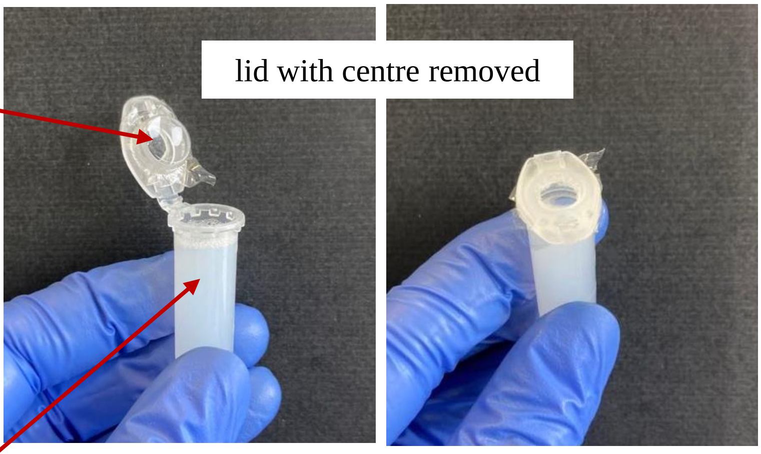

2 ml of nanoparticle suspension

2. Place epp tubes into floating microtube rack.

floating microtube rack

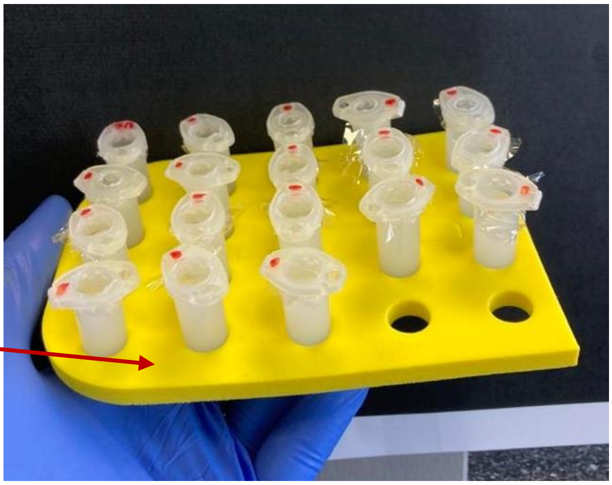

3. Place floating microtube rack with epps upside down into 1,2 l of dissolution buffer in water bath, 37 ˚C.

dissolution buffer 1,2 l

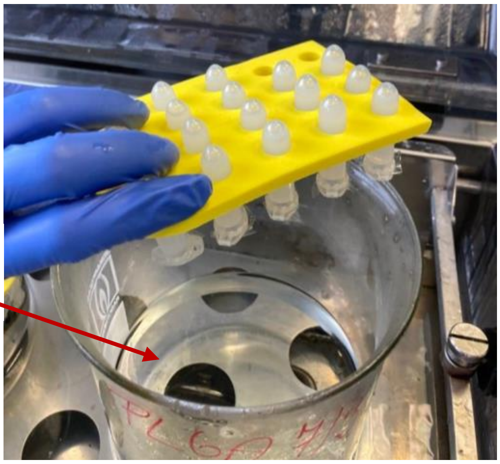

4. At predetermined time points remove desired number of epps and evaluate DLE%. Compare DLE% with DLE% of 0h point (nanoparticle suspension immediately after preparation). According decrease of DLE% the release of DA is calculated.

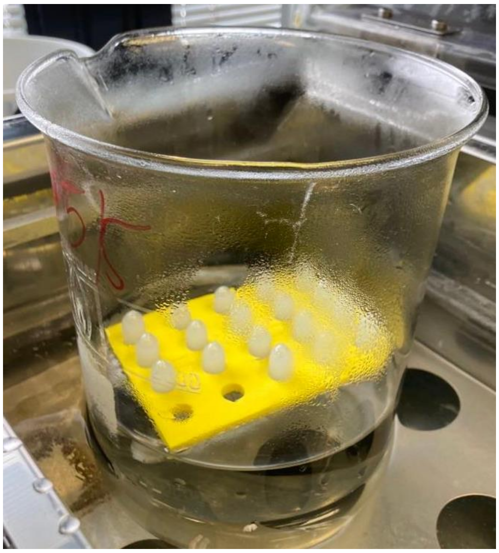

Supplement: Supplementary file 2 — Supporting Information [file MABI-25-2400411-s004.pdf]
